# Heterogeneity of early-onset conduct problems: assessing different profiles, predictors and outcomes across childhood

**DOI:** 10.1186/s13034-025-00897-2

**Published:** 2025-04-16

**Authors:** Beatriz Díaz-Vázquez, María Álvarez-Voces, Estrella Romero, Laura López-Romero

**Affiliations:** Institute of Psychology (IPsiUS), University of Santiago de Compostela, Santiago de Compostela, Spain

**Keywords:** Heterogeneity, Conduct problems, Oppositional, Callous-unemotional, Attention deficit/hyperactivity, Childhood

## Abstract

**Background:**

Among early-onset conduct problems (CP), associated with more disruptive behaviors of greater intensity and stability, several domains have been proposed from a variable-centered perspective to capture their heterogeneity: oppositional defiant disorder (ODD), attention-deficit/hyperactivity disorder (ADHD), and callous-unemotional traits (CU). Using a person-centered approach, the present study aims to identify distinct profiles of child CP, examining different predictors and developmental outcomes.

**Methods:**

Data included parent- and teacher-reported questionnaires from an ongoing longitudinal study (ELISA). Latent profiles were identified first in a community sample (*n* = 2,103; age 4–8 years; 50.9% boys) and replicated in a high-CP subsample (*n* = 168; 70.24% boys).

**Results:**

Four profiles emerged in the community sample (Normative Development, Daring/Impulsive, Low prosociality + Fear; Low prosociality + Psychopathic Traits [PP]), and three in the high-CP sample (same except the normative). The identified CP profiles aligned with the ADHD and CU domains, but not the ODD domain. Differences in activity, punitive and inconsistent parenting emerge as the most significant predictors. Regarding the outcomes, the Low prosociality + PP profile stands out as the group with the most severe emotional, social and behavioral maladjustment.

**Conclusions:**

These findings highlight the heterogeneity within CP, and the importance of designing specific and tailored interventions for each identified profile.

**Supplementary Information:**

The online version contains supplementary material available at 10.1186/s13034-025-00897-2.

## Introduction

Conduct problems (CP) are conceptualized as a recurring pattern of behavior that breaches the rights of others or age-appropriate norms and rules [1], representing the foremost reason for clinical [2] and psychoeducational assistance [3]. CP impact several critical domains of childhood development, including family and school problems [4, 5], and has consistently served as a long-term predictor of antisocial behavior problems in adolescents and adults [6], mental health problems [7] and school maladjustment [8]. Despite the social [2], legal [9], and economic burden [10] that CP entail, there is still a need to better understand the high heterogeneity and comorbidity associated with this behavioral spectrum.

### Heterogeneity of CP: pathways and profiles across childhood

CP can be present very early in life [11, 12] and are manifested in diverse forms that include differences at the phenotypic, etiological and developmental level, exhibiting a heterogeneous landscape across lifespan [13]. Developmentally, several studies have examined different trajectories from early childhood, evidencing at least three or four distinct CP trajectories across development, including normative or stable low, childhood-limited, adolescent-onset and persistent pathways [14, 15].

These results extend the initial classical theories that postulated the existence of two trajectories named early-onset or life-course-persistent, and late-onset or adolescence-limited antisocial behavior [16]. Among these, the early-onset group gathered specific attention, because of its elevated risk for later maladjustment [17] and the significant heterogeneity within this subgroup [18, 19]. In recent years, the role of sex has also started to be considered as a part of this heterogeneity. Thus, although the existence of early-onset CP in girls has been debated [20], recent studies have found girls exhibiting this trajectory [21, 22], but also suggesting that girls tend to desist from CP, particularly in their overt forms [23], earlier than boys [21].

An early approach to understanding the development of serious CP within the early-onset group established two main pathways, represented by two phenotypically distinctive subgroups: one characterized by cognitive and emotional regulation problems leading to impulsive acts of reactive aggression [24], and another characterized by the presence of callous-unemotional (CU) traits, present in a small but high-risk group of children with more severe and stable trajectories of misbehavior [19]. Accordingly, both emotional dysregulation, including irritability as a core temperamental trait, and CU traits, considered as the extreme pole of low prosociality, were underscored as key indicators of CP heterogeneity [25, 26], and proved usefulness in the identification of different profiles of child CP [27]. Expanding this distinction, Waller et al. [28] postulated a more comprehensive proposal identifying three different domains characterized by oppositional (ODD), attention-deficit/hyperactivity disorder (ADHD) and CU behaviors, with ODD and CU domains largely converging with the previous dual conceptualization [24]. These domains could be organized into two distinctive pathways: The “hot” pathway would be characterized by emotional and behavioral dysregulation [18], and it would include children with *ODD* behavior, exhibiting negative emotionality, elevated internalizing symptoms and anger regulation difficulties [29], and children with *ADHD* symptomatology, characterized by poor inhibitory control, deficits in attention and impulsivity [30]. The “cold” pathway, on the other hand, would be associated with *CU* behaviors, marked by uncaring, remorseless behaviors, deficits in empathy, high proactive aggression and deficits in conscience development [31]. This theoretical distinction was supported by studies where items from the Child Behavior Checklist (CBCL [33]) formed separate scales of ADHD, ODD and CU behaviors at age 3 [33, 34]. These domains were later validated longitudinally, with CU, ODD and ADHD behaviors showing differential associations with socioemotional, cognitive and behavioral outcomes [28].

### Delving into the heterogeneity of CP

#### Risk and protective factors

Genes and environment act, through multiple individual and contextual mechanisms [35], as a determinant for psychopathology [36]. At the *individual* level, child temperament is a key factor in the development of CP. The aforementioned multiple pathways to early-onset CP largely converge, at the dispositional level, with the developmental propensity model to CP [37], including oppositional temperament (e.g., negative affect), inhibited harm avoidance (e.g., daring), and low prosociality (e.g., CU traits) as temperamental contributions of antisocial propensity [38]. Relatedly, traits such as impulsivity [39], negative affect, and emotional dysregulation [40, 41] form an undercontrolled profile, associated with both short- [42] and long-term [43] externalizing behaviors. Although these frameworks emphasize some common factors for childhood psychopathology [44] recent studies call for a more nuanced distinction of temperamental traits underlying CP. In this regard, low sensitivity to threat (fearlessness) has been linked specifically to the CU domain [45, 46], while irritability, a core temperamental trait in CP development [26], is particularly associated with the hot pathway and ODD behavior [28, 47].

In addition to these temperamental and individual factors there is another set of variables that have been related to CP across time. *Parental practices* have been widely studied as predictors of future positive or maladjusted behavior, often in interaction with child characteristics [45]. Parental warmth, the foundation of positive parenting, characterized by a high degree of positive affection, dedication, and a sense of closeness to children [48, 49], has been established as a protective factor against the future occurrence of CP [50, 51]. In contrast, punitive parenting, marked by harshness and punishment, has been linked to the future development of externalizing behavior problems during childhood across several longitudinal studies [52, 53] and, more specifically, with a significant association to the CU group [54]. Currently it is also known that children’s temperamental characteristics can influence parental style, with a notable relationship observed in the dyad fearlessness—ineffective parental practices (i.e., low warmth and harsh punishment) [55], confirming that individual and environmental factors tend to be in constant interaction.

#### Later outcomes

Although each of the above-mentioned domains (ODD, ADHD and CU behaviors) on their own have sometimes been associated with poor adjustment later in adulthood, when they are combined with high levels CP, the prognosis tends to be worse for multiple types of problem behaviors in adolescence and early adulthood, including violent behavior [56, 57].

Children showing the combination of CU traits and CP (CU + CP profile) usually show worse prognosis later in development, characterized by lower empathy, prosociality and more severe and persistent antisocial behavior [31, 58], bullying [59, 60] and lower academic achievement [61]. However, this combination (CU + CP) has recently been refined by incorporating the full constellation of psychopathic traits (i.e., grandiose-deceitful [GD], CU, and impulsive-need of stimulation [INS] [62]), not only to better inform the heterogeneity of CP, but also to predict more severe CP [63, 64] and other related variables, including ADHD symptoms [65] and aggression [66]. Related to aggression, in school environment, bullying has gained special relevance, associated with externalizing behaviors [67] and with the CU profile especially [59]. The victim profile has been particularly associated with internalizing behaviors [67] but also recently with the CU profile [68].

### Current study

Recent studies have emphasized the heterogeneity within the child and youth population, particularly among children with CP. Theoretical frameworks have linked various behaviors to distinct domains (e.g., ODD, ADHD, and CU) demonstrating their relevance from variable-centered approaches [33, 34]. Over time, these associations have been tested in longitudinal studies with clinical [28] and at-risk samples [54]. However, a frequently cited limitation was the need for a person-centered approach, which is particularly beneficial when studying complex and heterogeneous phenomena like CP [69]. Assuming a person-centered approach provides distinct advantages over the variable-centered approach such as the ability to account for individual differences while maintaining some degree of homogeneity, offering greater accuracy and parsimony in capturing the complexities inherent in these behaviors [70]. Starting from this perspective of analysis and using a community sample, the present study is the first to attempt to replicate the existence of the three previously mentioned domains by: (1) Identifying CP profiles based on temperamental and personality variables (e.g., negative emotionality, prosociality, daring, GD, CU, INS, fearlessness) across childhood in both community and high-CP samples; (2) exploring individual and family-level variables that predict profile membership; and (3) examining developmental outcomes related to these profiles. In line with the latest classifications addressing heterogeneity and its associated domains, our main hypothesis was to identify three distinct groups within the CP subsample, following the model of Waller et al. [28]. Similarly, we expected the existence of these three groups within the total sample, anticipating the emergence of a fourth group—normative and majority—given the community-based nature of the sample.

## Method

### Participants

The present study used data from the longitudinal ELISA project (*Estudio Longitudinal para una Infancia Saludable*), carried out in Galicia (NW Spain). In this specific research, five waves of the ELISA project, original covering a six-year data collection period, were used to address the different objectives proposed in this study. Parent-reported data from wave 3 (T3: 2019; *n* = 2,105; 50.9% boys; *M*age = 6.10, *SD* = 0.93, range = 4–8 years) was employed for identifying CP profiles in both the total sample (*n* = 2,103; two cases deleted due to complete missing data on latent profile indicators) and a high CP subsample (*n* = 168; 70.24% boys). The High CP group was identified post-hoc from the community-based sample. To identify this subsample, a cut-off point of 1.5 *SD* was used to determine the presence and intensity of CP above the mean. Children (93.9% Spanish) were attending 72 different schools (76.4% public, 20.8% charter and 2.8% private) located in predominantly working-class communities from different urban, suburban and rural areas. At T3, 82.5% of mothers and 93.3% of fathers were actively working. Additionally, we used parent-reported data from wave 1 (T1: 2017; *n* = 2.266; *M*_age_ = 4.26, *SD* = 1.02, range = 3–6 years) to examine different temperamental and family predictors, as well as data from wave 4, (T4: 2021; *M*_age_ = 8.21, *SD* = 1.17, range = 6–10 years), wave 5 (T5: 2022; *M*_age_ = 7.83, *SD* = 1.04, range = 7–10 years) and wave 6 (T6: 2023; *M*_age_ = 10.24, *SD* = 1.05, range = 8–12 years) to examine developmental outcomes reported by parents (*n* = 1,291, 1,630 and 1,343 from T4 to T6, respectively) and teachers (*n* = 1,426 and 1,675 for T4 and T5 respectively; no teacher-reports were collected in T6). Of note, in 2018 (T2) the initial sample was increased by 361 participants (51.5% boys, aged 3 to 5; *M*_age_ = 3.77; *SD* = 0.87) from a specific area within the same region not covered in T1. As commonly observed in longitudinal studies, attrition across waves was mostly derived from participants’ withdrawal, lack of success in additional contacts for a follow-up participation, by non-returning the questionnaire, or even for mortality or frailty [71].

### Instruments

The main informants for this study were parents/caregivers (87.3% mothers). Teachers’ reports were also used to assess certain developmental outcomes. A list of the variables used in the study (see Table S1) along with their descriptive statistics (Table S2) can be consulted in the Appendix.

#### Latent profile indicators (T3)

##### Parent-reported

*Children’s temperamental variables.* Negative emotionality (e.g., “Does your child react intensely when he/she gets upset”; α = 0.77), daring (e.g., “Is he/she daring and adventurous?” α = 0.83) and prosociality (e.g., “Spontaneously shares” α = 0.78) were explored using the Child and Adolescent Dispositions Scale (CADS-P [72]), adapted from Mathesius et al. [73]. This instrument consists of 12 items coded on a 5-point Likert scale ranging from 1 (*none*) to 4 (*totally*).

*Children’s Psychopathic Traits.* Children’s psychopathic traits were examined using the Child Problematic Traits Inventory [CPTI] [63]. This instrument consists of 28 items with a 4-point Likert scale ranging from 1 (*does not apply at all*) to 4 (*applies very well*). Eight items were used to measure GD (e.g., “Lies often to avoid problems”; α = 0.83), 10 to measure CU (e.g., “Often does not seem to care about what other people feel and think”; α = 0.88) and 10 to measure INS (e.g., “Often has difficulties with awaiting his or her turn”; α = 0.86).

*Fearlessness.* Six items (e.g., “He/she does not seem to be afraid of anything”; α = 0.87) coded on a 4-point Likert scale ranging from 1 (*does not apply at all*) to 4 (a*pplies well*) were utilized for evaluating fearlessness [63].

*Conduct Problems*. The Conduct Problems Scale [63], a 10-item measure intended to assess symptoms from ODD and conduct disorder (e.g., “Threatens others”; α = 0.87) was used to identify a subsample of children with High CP (1.5 *SD* above the mean; *n* = 168) for replication purposes. Items were rated with a 5-point Likert-type scale ranging from 1 (*never*) to 5 (*almost always*).

#### Additional variables for profile further definition (T3)

*Child behavioral variables.* Six items for oppositional defiant (e.g., “Disobeys parents”; α = 0.76), seven items for attention deficit/hyperactive (e.g., “Can’t concentrate, can’t pay attention for long”; α = 0.80) and nine items for anxiety problems (e.g., “Worries a lot”; α = 0.70) were used from the Child Behavioral Checklist 6 -18- DSM-Oriented Scales (CBCL [32]).

#### Predictor variables (T1)

##### Parent reported

*Children’s temperamental variables.* Emotionality (five items, e.g., “Cries easily”; α = 0.71), sociability (Four items, e.g., “Likes to be with people”; α = 0.50^1^), shyness (three items, e.g., “Tents to be shy”; α = 0.75) and activity (three items, e.g., “Is off and running as soon as he/she wakes up in the morning”; α = 0.83) were explored using the Spanish adaptation of EAS, Temperament Survey for Children (EAS TS-C [75]). This instrument consists of 15 items from four subscales, coded on a five-point Likert scale ranging from 1 (*not characteristic of my child*) to 5 (*very characteristic of my child*).

*Social competence.* Prosocial/Communication skills (six items, e.g., “Your child listens to others’ points of view”; α = .81) and emotion regulation (six items, e.g., " Your child resolves problems with friends or brothers and sisters on his/her own”; α = 0.80) were evaluated via the FastTrack scale [76] with 12 items coded on a 5-point Likert scale ranging from zero (*not at all*) to 4 (*very well*).

*Parental warmth.* Parental warmth was measured by six items based on the Child Rearing Scale [77]. The items (e.g., “We shared pleasant and loving moments together”; α = 0.82) had a 5-point Likert-type response scale ranging from 1 (*never*) to 5 (*always*).

*Parent practices.* Different parent practices were measured using the Spanish-adapted form of the Alabama Parenting Questionnaire- Preschool revision (APQ-Pr [78]). This instrument has three subscales: positive practices (12 items, e.g., “Friendly talk with your child”; α = 0.75), inconsistent practices (7 items, e.g., “Threaten to punish your child and then do not punish”; α = 0.68) and punitive practices (5 items, e.g., “Spank your child with hand when something wrong”; α = 0.52). The APQ-Pr has 24 items coded on a five-point Likert scale ranging from 1 (n*ever*) to 5 (a*lways*).

#### Developmental outcomes (T4-T6)

##### Parent reported (T4-T6)

*Children’s CP.* Parents rated the aforementioned the Conduct Problems Scale [63] to assess the development of CP in T4 (α = 0.88), T5 (α = 0.87) and T6 (α = 0.86).

*Behavioral and psychosocial adjustment.* The Strengths and Difficulties Questionnaire (SDQ [79]) was used to assess: hyperactivity (e.g., “Restless, overactive, cannot stay still for long”; α = .80, .81, .81, for T4, T5 and T6 respectively), emotional symptoms (e.g., “Often unhappy, downhearted or tearful”; α = .71, .71, .71), peer problems (e.g., “Rather solitary, tends to play alone”; α = .63, .63, .65), and prosocial behavior (e.g., “Considerate of other people’s feelings”; α = .65, .65, .66). Items (five per scale) were coded on a three-point Likert scale ranging from 1 (n*ot true*) to 2 (c*ertainly true*).

*Bullying/victimization.* An adaptation with two subscales of Barker et al. [80] was employed. This measure consists of two subscales with four items each: bullying (e.g., “He/she hits or pushes other children”; α = 0.72, 0.67, 0.75) and victimization (e.g., “Hit or pushed by other children”; α = 0.89, 0.89, 0.90). Response options ranged from 1 (n*ever*) to 5 (a*lmost always*) in a five-point Likert-scale.

##### Teacher-reported (T4-T5)

Teachers provided information on longitudinal outcomes at T4 and T5. They rated the same aforementioned scales, including scores on CP (α = 0.93, 0.92, at T4 and T5 respectively), hyperactivity (α = 0.86, 0.85), emotional symptoms (α = 0.74, 0.73), peer problems (α = 0.69, 0.71), prosocial behavior (α = 0.77, 0.80), bullying (α = 0.86, 0.84) and victimization (α = 0.85, 0.87).

### Procedure

The longitudinal ELISA project^2^, initiated in 2017, is an ongoing study that has been continuously conducted up to the present day. The research study and procedures were reviewed and approved by Bioethical Committee at the Universidade de Santiago de Compostela: Approval Code: USC-21/2020; Approval Date: June 17th 2016, and November 9th 2020. A convenience sampling approach was used to initially contact 126 schools, of which 72 agreed to participate in the study (public, charter and private). Families were contacted to be invited to participate. After asking for their formal consent, they had one month to complete a questionnaire (paper or online format) at each wave of data collection. Reminders were sent by calls or e-mail by the ELISA staff. The same procedure was followed for teacher respondents. Data collection took place during spring, to ensure that teachers had spent at least six months with children before rating questionnaires. Neither teachers nor parents received any monetary compensation for their participation. Instead, parents and schools were rewarded throughout the different data collection. Schools received educational games for children in T1 and T2. A draw of several sets of books and educational games, valued between EU 50 and 100, was carried out at the end of T3 for both families and schools. At T4, T5 and T6, parents received a report of results about their child’s competencies, with suggestions for improvement, based on their responses to the questionnaires. Additionally, formative talks were offered to teachers and families upon request during all study waves. Confidentiality was ensured through pseudo-anonymity, with each participant assigned a unique ID and alphanumeric code for secure questionnaire access.

### Statistical analyses

First, to examine the potential nested nature of the sample, the intraclass correlation coefficient (ICC) at the school level was calculated in IBM SPSS Statistics 28.0 for each subscale to be used in the subsequent Latent Profile Analysis (LPA) and to classify participants as High CP. The ICC values were 0.02 or lower, indicating minimal clustering. Since substantial clustering is generally considered when ICC values exceed 0.05 [81], we concluded that accounting for nesting within schools was not required for the analysis of this study.

Second, The Little’s Missingness Completely at Random (MCAR) test revealed that attrition was not missing completely at random, χ^2^ (236) = 2375.19, *p* = .025 [82]; yet, the normed test (χ₂ / df) was 1.04, which is below the suggested cut-off of 2, indicated that data was Missing at Random (MAR) [83]. Subsequently, LPA was conducted in Mplus v 7.4 [84] to identify distinct latent profiles of children based on different temperamental and personality variables. To handle missing data, we employed the full information maximum likelihood (FIML) estimator for profile analyses. This method has been shown to provide unbiased parameter estimates, particularly when compared to deletion-based techniques (e.g., listwise deletion) [85], and is especially effective in addressing random data loss and higher rates of missing data [86]. Different models specifying varying numbers of latent profiles were tested. Statistical criteria, along with theoretical and clinical relevance, were used to compare models and identify the optimal number of profiles. Differences in latent profiles indicators were examined with ANOVA in IBM SPSS Statistics 28.0. Subsequently, the Auxiliary option in Mplus was used to compare the best fitting solution on theoretically relevant cross-sectional correlates (BCH method) and early predictors (R3STEP method). This approach was preferred over traditional analyses (e.g., logistic regression) because it allows to examine how auxiliary variables differed across profiles without influencing the classification process whilst takes participant`s partial membership into account [87]. Finally, to test differences on longitudinal outcomes, repeated measures ANOVA with Bonferroni post-hoc test comparison were performed in SPSS. These analyses were conducted analogously, following the same steps in both analyzed samples. First, the analyses were performed on the total sample and then replicated in the High CP subsample.

## Results

### Model fitting and profile selection

The BIC is a key criterion for comparing latent profile models, with lower values preferred [88]. However, the theoretical interpretability of the profiles is also crucial [89]. For the total sample, although the 5-profile model had lower values of AIC, BIC and ABIC, than the 4-profile model, the LMR test was not significant (*p* = .364), indicating no significant improvement when increasing the number of latent profiles [90]. Entropy values (better accuracy in classifying individuals into latent profiles) were slightly higher for the 5-profile model (0.91) compared to the 4-profile model (0.89), but the difference was not substantial and still represents accurate classification. As reinforcement for this decision, we have prioritized theoretical interpretability aligning with the idea that a solution with superior statistical fit indices is not meaningful if it lacks theoretical coherence [89]. Hence, the four-class solution better represents the hypothesized profiles, while the addition of another profile did not contribute significantly to the model`s interpretability, as the emergence of a distinctive profile was lacking. Considering both statistical and theoretical criteria, we favored the *4-profile model* over the 5-profile solution as it provides a more parsimonious solution to our data.

For the High CP group both statistical fit indices and theoretical/clinical usefulness were also considered to select the best model. Although the LMR test did not indicate a significant improvement with the addition of a third profile, the entropy value (0.76) was similar, and the AIC and BIC indices were lower, suggesting a better balance between fit and complexity [91]. For this group, literature also supports the three-profile model, as it offers a more detailed classification consistent with the complexity of CP-related variables, whilst adding one more profile did not favor interpretability. Therefore, the *3-profile model* was chosen over the 4-profile solution due to its greater descriptive clarity, practical relevance, and parsimony [92].

Model fit indices of LPA in total sample and High CP subsample can be found in Table 1.

**Table 1:** Model fit indices from latent profile analysis (LPA) in total sample and high CP subsample

		Entropy	AIC	BIC	ABIC	LMR (*p*)	BLRT (*p*)
Total sample	1 Class	–	41608.87	41687.99	41643.51	–	–
	2 Class	0.86	38096.97	38221.29	38151.39	< 0.001	< 0.001
	3 Class	0.89	36734.31	36903.84	36808.53	< 0.001	< 0.001
	4 Class	0.89	35786.59	36001.33	35880.60	0.005	> 0.01
	5 Class	0.91	35318.18	35578.13	35431.98	0.364	< 0.001
High CP 1.5 SD	1 Class	–	3626.76	3670.50	3626.17	–	–
	2 Class	0.76	3491.02	3559.75	3490.09	0.02	< 0.001
	3 Class	0.76	3426.27	3519.99	3424	0.30	< 0.001
	4 Class	0.82	3369.88	3488.59	3368.28	0.32	< 0.001

AIC = Akaike Information Criterion; BIC = Bayesian Information Criterion; ABIC = Adjusted Bayesian Information Criterion; LMR = Lo, Mendell and Rubin likelihood ratio test; BLRT = Bootstrap Likelihood Ratio Test; CP = Conduct Problem; SD = Standard Deviation

Differences between profiles (both in total sample and High CP group) on latent profile indicators were confirmed with ANOVA tests (all remained significant at *p* <.05). Comparisons between groups are shown in Table 2 respectively.

**Table 2:** Comparisons across TS and high CP profiles on latent profiles indicators (T3)

	Total sample						High CP				
	Normative development	Daring / impulsive	Low prosociality + fear	Low prosociality + PP	F^b^(df = 3)	η^2^	Daring / impulsive	Low prosociality + fear	Low prosociality + PP	F^b^(df = 2)	η^2^
	M^a^ (SD)	M (SD)	M (SD)	M (SD)			M (SD)	M (SD)	M (SD)		
Negative emotionality	− 0.37 (0.02)_a_	0.37 (0.05)_b_	0.37 (0.05)_b_	1.22 (0.08)_c_	222.68***	0.24	1.42 (0.13)_a_	1.24 (0.11)_a_	2.05 (0.15)_b_	9.522***	0.10
Prosociality	0.33 (0.02)_c_	0.28 (0.04)_c_	− 0.89 (0.05)_b_	− 1.30 (0.08)_a_	358.98***	0.34	0.10 (0.10)c	− 0.69 (0.11)_b_	− 1.78 (0.14)a	52.568***	0.39
Daring	− 0.50 (0.02)_a_	1.38 (0.03)_d_	− 0.25 (0.03)b	1.16 (0.07)_c_	1034.25***	0.60	1.51 (0.10)b	− 0.16 (0.08)a	1.39 (0.14)b	93.840***	0.53
GD	− 0.43 (0.02)_a_	0.01 (0.04)_b_	0.73 (0.05)_c_	1.73 (0.11)_d_	505.40***	0.42	0.73 (0.14)_a_	1.19 (0.14)_a_	2.11 (0.26)_b_	13.873***	0.14
CU	− 0.54 (0.01)_a_	− 0.13 (0.03)_b_	1.05 (0.04)_c_	2.22 (0.08)_d_	1749.47***	0.71	0.61 (0.10)_a_	0.85 (0.13)_a_	2.93 (0.15)_b_	77.839***	0.49
INS	− 0.50 (0.02)_a_	0.63 (0.05)_b_	0.48 (0.04)_b_	1.33 (0.06)_c_	432.82***	0.38	1.28 (0.10)_a_	0.61 (0.10)_b_	1.76 (0.10)_c_	33.30***	0.29
Fearlesness	− 0.56 (0.01)_a_	1.28 (0.04)_c_	− 0.05 (0.03)_b_	1.49 (0.07)_d_	1228.03***	0.64	1.56 (0.11)b	− 0.11 (0.07)a	0.89 (0.09)b	94.066***	0.53

TS = Total Sample; CP = Conduct Problems; PP = Psychopathic Traits; M = Mean Z score; SD = Standard Deviation; GD = Grandiose-Deceitful; CU = Callous-Unemotional; INS = Impulsive-Need for Stimulation. Different subscripts (a, b, c, d) refer to significant differences between classes (*p* <.05) with post hoc Bonferroni test for multiple pairwise comparisons

^a^Mean values represent overall estimates of each analyzed variable across profiles

^b^*F* values represent between-subjects effect tests

***p <.001

**Fig. 1:**
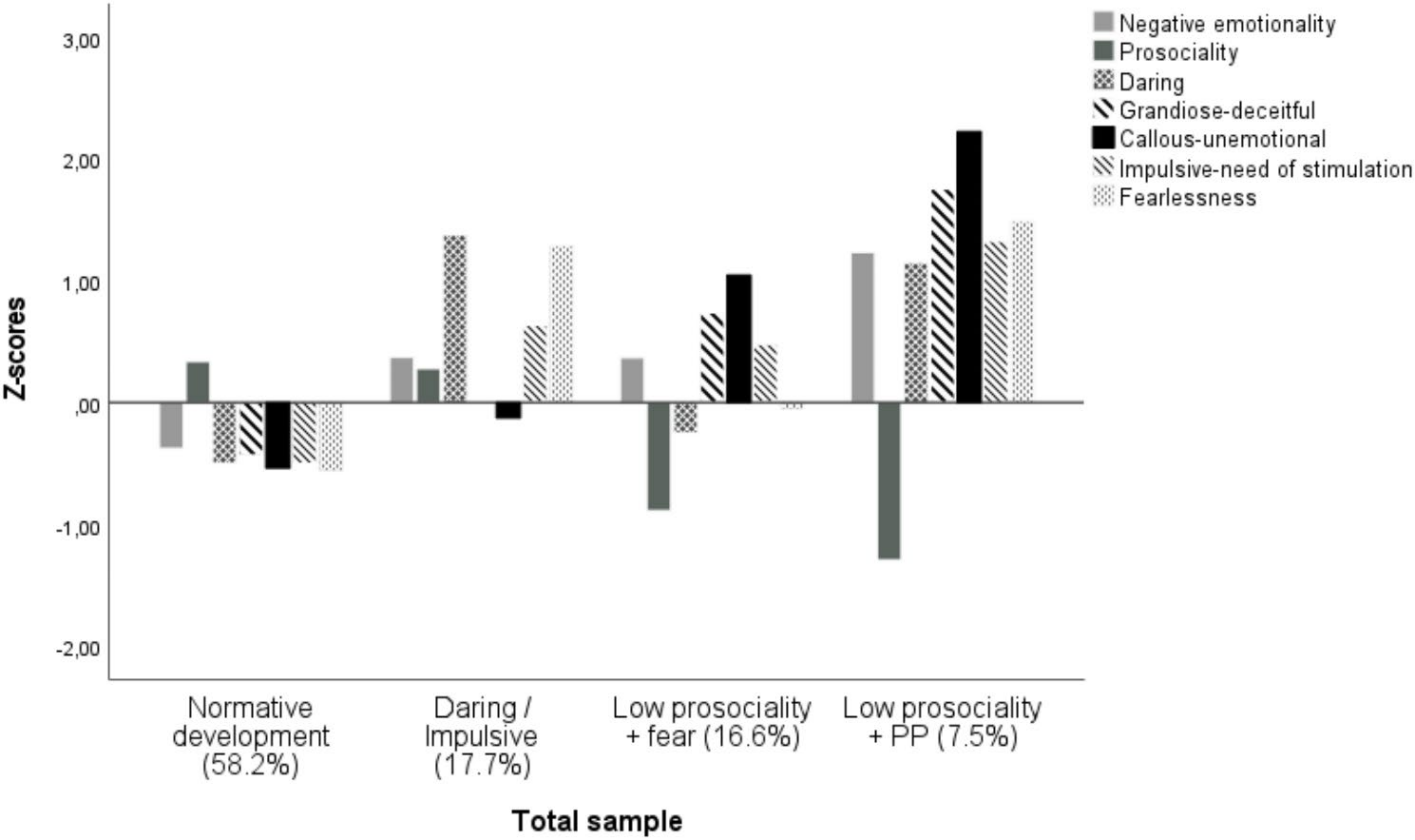
Groups identified using Latent Profile Analysis at T3 (ages 4–8) in total sample (*N* = 2,103)

**Fig. 2:**
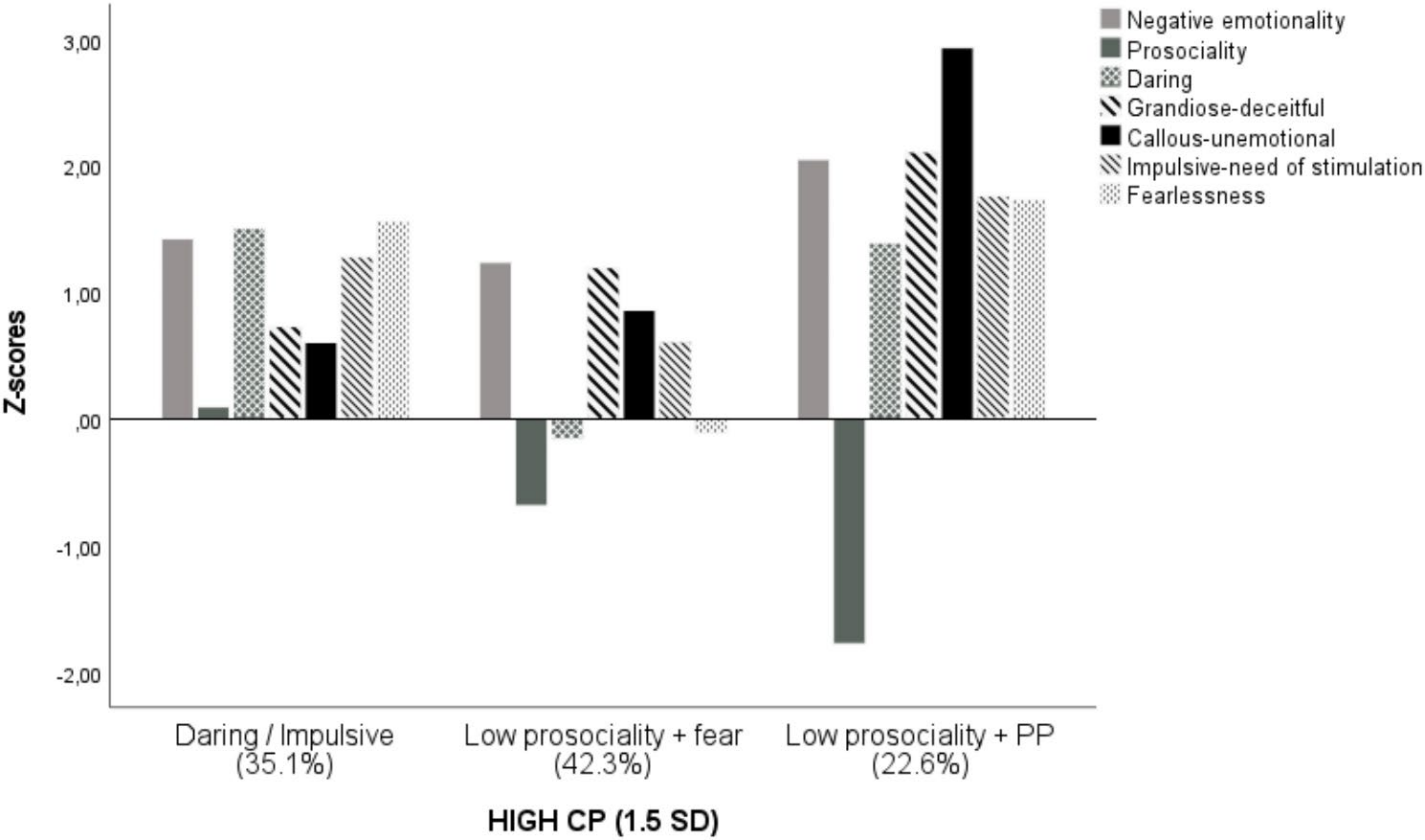
Groups identified using Latent Profile Analysis at T3 (ages 4–8) in the High CP group (1.5 *SD*; *N* = 168)

Representations of identified profile solutions, based on mean standardized *z*-scores for latent profiles indicators, are shown in Figs. 1 and 2 (for total sample and High CP subsample respectively). Both the Z-scores of the total sample and those of the High CP group were computed in the T3 community sample. Four profiles emerged from the total sample, with the *Normative development profile* standing for several reasons, including the number of children it encompasses (*n* = 1,224; 45.9% boys), their significant differences in all variables with all other groups (exception of prosocial behavior which does not differ from the daring-impulsive profile), and its below-average scores in nearly all the variables analyzed (approximately 0.5 *SD* below), with the sole exception of prosocial behavior, in which this group achieved the highest score. The second largest profile (*n* = 373; 17.7%; 56.8% boys) was the *Daring/Impulsive profile*, which, along with the Normative development profile, was the only one to score above the mean in prosocial behavior, with no significant differences between them. This profile stood out especially for a marked daring, with more than 1*SD* above average. Finally, we found two profiles characterized by low prosociality. First, the *Low prosociality + fear profile* (*n* = 349; 16.6%; 57.3% boys), characterized by low scores in prosociality but also low average scores in daring and fearlessness. Of note, no significant differences were observed with the Daring/Impulsive profile on negative emotionality and INS traits. Second, the *Low prosociality + PP Profile* (*n* = 157; 7.5%; 61.8% boys) showed the lowest score in prosociality (more than 1SD below average) and significantly higher scores in negative emotionality, psychopathic traits, especially CU traits, and fearlessness compared with all groups.

In the High CP group (*n* = 168; 70.2% boys), only three profiles emerged: *Daring/impulsive* (*n* = 59; 35.1%; 62.7% boys), Low prosociality + fear (*n* = 71; 42.3%; 74.6% boys); Low prosociality *+ PP* (*n* = 38; 22.6%; 73.7% boys). In all three groups, as a rule, all the variables analyzed scored above the mean, except for prosocial behavior, with scores below the mean for the low prosociality + fear (1*SD*) and, remarkably, the Low prosociality + *PP* (2*SD*). Low prosociality + *PP* was the smallest group, but with the highest scores across all variables compared with all remaining groups except for daring and fearlessness (no differences with daring-impulsive profile). Differences between groups on latent profile indicators were not as clear as observed for the total sample. Thus, there were no significant differences between Daring/impulsive and Low prosociality + fear profile on emotional reactivity, GD and CU traits. These groups only remained different in prosociality and INS (lower for Low prosociality + fear group). Differences between Daring/impulsive and Low prosociality + *PP* were significant for all variables except daring and fearlessness.

Taking both samples into consideration (see Table 2), several observations can be made. First, the Normative development profile (the only one that included more girls than boys) did not emerge in the high CP group, as could be expected. The Low prosociality + *PP* group clearly manifested in both groups (total sample and High CP subsample) as the most minor, but with more extreme values in the analyzed variables. The Low prosocial + fear profile showed the same trend in both samples, with below-average scores in prosocial behavior, daring, and fearlessness. Finally, the Daring/Impulsive group did not emerge in the same way across both samples (i.e., the values of the variables did not follow exactly the same trends in terms of positive or negative scores, nor in terms of intensity) but both profiles showed a clear shared tendency to score higher in daring, INS traits and fearlessness.

### Further definition of profiles in behavioral variables (T3)

To better understand the characteristics of each resulting group, several behavioral variables (i.e., ODD, ADHD, and Anxiety; [CBCL [32]]) central in the definition of CP [28] were analyzed (see Table 3). All of them remained significant (*p* <.05). In the total sample, the normative development group showed the lowest scores across variables. Significantly higher scores in all ODD, ADHD and Anxiety symptoms were observed for the Low prosociality + *PP* group. No significant differences were observed between the normative development profile and the Daring/Impulsive group in terms of anxiety, nor between the daring/impulsive and Low prosociality + fear groups in ODD.

**Table 3:** Cross- sectional comparisons between TS and high CP profiles on behavioural variables parent reported (T3)

	Total sample						High CP				
	Normative development	Daring / impulsive	Low prosociality + fear	Low prosociality + PP	X²	η^**2**^	Daring / impulsive	Low prosociality + fear	Low prosociality + PP	χ²	η^**2**^
	M ^a^ (SD)	M (SD)	M (SD)	M (SD)			M (SD)	M (SD)	M (SD)		
ADHD	0.35 (0.01)_a_	0.77 (0.02)_c_	0.63 (0.03)b	1.08 (0.04)_d_	212.49***	0.24	1.04(0.06)_b_	0.77 (0.06)_a_	1.36 (0.06)_c_	46.71*	0.21
ANX	0.35 (0.01)_a_	0.37 (0.02)_a_	0.45 (0.02)_b_	0.54 (0.03)_c_	19.96***	0.03	0.53 (0.05)_a_	0.59 (0.05)_b_	0.68 (0.06)_c_	3.08*	0.11
ODD	0.29 (0.01)_a_	0.65 (0.02)_b_	0.61 (0.02)_b_	0.98 (0.04)_c_	226.30***	0.25	1.10 (0.05)b	0.98 (0.05)_b, a_	1.24 (0.07)_a, c_	8.78*	0.06

TS = Total Sample; CP = Conduct Problems; PP = Psychopathic Traits; M = Mean Z score; SD = Standard Deviation; ADHD = Attention-Deficit/Hyperactivity-Disorder, ANX = Anxiety; ODD = Oppositional Defiant Disorder. Different subscripts (a, b, c, d) refer to significant differences between classes (*p* <.05) with post hoc Bonferroni test for multiple pairwise comparisons

^a^Mean values represent overall estimates of each analyzed variable across profiles

*p <.05 ***p <.001

Overall, for the High CP subsample, higher values were observed across all groups and variables compared to the corresponding groups in the total sample (see Table 3). Notably, the Low prosociality + *PP* showed again the highest values in all three variables: ODD, ADHD (both followed by the daring/impulsive group), and Anxiety (followed by the Low prosociality + fear group). All groups differed from each other across all variables except for one pair: Low prosociality + fear and Low prosociality + *PP* in ODD.

### Predictor variables for the different profiles (T1)

As predictor variables for classification in each profile, two main blocks were used: variables related to *parenting* style (i.e., warmth, punitive, inconsistent and positive parenting) and *individual* variables (i.e., emotionality, sociability, shyness, activity and social competence).

In the total sample (see Table 4), higher levels of activity, punitive parenting, and inconsistent parenting, along with lower sociability, prosocial and communication skills, and emotional regulation, predicted membership in the Low prosociality + *PP* group compared to the normative development group. This Low prosociality + *PP* group also differed from the daring/impulsive group in a more punitive parenting style and lower prosocial and communication skills, while higher levels of activity were the only factor distinguishing it from the Low prosociality + fear group. Lower shyness, more activity, and greater prosocial and communication skills predicted membership in the Daring/Impulsive group compared to the Low prosociality + fear group. What differentiated the normative development group from the Daring/Impulsive group was greater emotional regulation, higher shyness, and lower activity. When comparing the Normative development group with the Low prosociality + fear group, we found that better communication skills, less punitive and more parental warmth were the key factors that distinguish membership in the normative group.

**Table 4:** Probability of belonging to different profiles based on temperamental and parenting variables in total sample (T1)

Comparison TS profile	Reference total sample profile		
	Normative development	Daring / impulsive	Low prosociality + Fear
	Est. (SE)	Est. (SE)	Est. (SE)
*Daring / Impulsive*			
Shyness	− 0.34 (0.08)***	–	
Activity	0.89 (0.11)***	–	
Emotionality	0 (0.12)	–	
Sociability	0.08 (0.15)	–	
Emotion regulation	− 0.71 (0.20)***	–	
Prosocial & communicative skills	0.07 (0.15)	–	
Positive parenting	− 0.28 (0.29)	–	
Inconsistent parenting	0.26 (0.17)	–	
Punitive parenting	0.38 (0.22)	–	
Warmth	− 0.23 (0.23)	–	
*Low prosociality + Fear*			
Shyness	− 0.09 (0.08)	0.25 (0.10)*	–
Activity	0.13 (0.10)	− 0.77 (0.14)***	–
Emotionality	0.07 (0.12)	0.07 (0.14)	–
Sociability	− 0.24 (0.16)	− 0.32 (0.19)	–
Emotion regulation	− 0.29 (0.19)	0.42 (0.25)	–
Prosocial & communicative skills	− 0.89 (0.16)***	− 0.96 (0.19)***	–
Positive parenting	− 0.22 (0.30)	0.06 (0.35)	–
Inconsistent parenting	0.34 (0.18)	0.08 (0.22)	–
Punitive parenting	0.79 (0.23)*	0.41 (0.27)	–
Warmth	− 0.51 (0.25)*	− 0.29 (0.27)	–
*Low prosociality + PP*			
Shyness	− 0.18 (0.11)	0.15 (0.12)	− 0.10 (0.12)
Activity	0.82 (0.15)***	− 0.07 (0.18)	0.69 (0.17)***
Emotionality	0.31 (0.16)	0.32 (0.18)	0.24 (0.18)
Sociability	− 0.44 (0.20)*	− 0.52 (0.22)*	− 0.20 (0.21)
Emotion regulation	− 0.68 (0.33)*	0.03 (0.36)	− 0.39 (0.35)
Prosocial & communicative skills	− 1.24 (0.26)***	− 1.31 (0.28)***	− 0.35 (0.28)
Positive parenting	− 0.03 (0.40)	0.26 (0.44)	0.20 (0.44)
Inconsistent parenting	0.67 (0.28)*	0.41 (0.30)	0.33 (0.31)
Punitive parenting	1.13 (0.30)***	0.74 (0.33)*	0.34 (0.34)
Warmth	− 0.29 (0.41)	− 0.06 (0.37)	0.23 (0.39)

SE = Standard Error; PP = Psychopathic Traits

*p <.05. ***p <.001

In the subsample of High CP, the Daring/Impulsive profile was associated with significantly more inconsistent parenting and activity compared to the Low prosociality + fear profile. The remaining variables were not significant in any other group comparisons (see Table 5).

**Table 5:** Probability of belonging to different profiles based on temperamental and parenting variables in high CP subsample (T1)

Reference CP profile		
	Daring / Impulsive	Low prosociality + Fear
	Est. (SE)	Est. (SE)
*Low prosociality + Fear*		
Shyness	0.58 (0.44)	–
Activity	− 1.85 (0.74)*	–
Emotionality	0.49 (0.57)	–
Sociability	− 0.40 (0.97)	–
Emotion regulation	0.04 (0.81)	–
Prosocial & communicative skills	− 0.13 (0.72)	–
Positive parenting	0.51 (1.40)	–
Inconsistent parenting	− 1.63 (0.83)*	–
Punitive parenting	1.45 (1.46)	–
Warmth	0.14 (1.38)	–
*Low prosociality + PP*		
Shyness	0.11 (0.34)	− 0.473 (0.330)
Activity	− 1.39 (0.76)	0.460 (0.448)
Emotionality	0.20 (0.70)	− 0.290 (0.466)
Sociability	− 0.80 (0.88)	− 0.400 (0.536)
Emotion regulation	− 0.21 (0.81)	− 0.251 (0.767)
Prosocial & communicative skills	− 0.88 (0.74)	− 0.747 (0.643)
Positive parenting	0.33 (1.02)	− 0.177 (1.180)
Inconsistent parenting	− 1.05 (0.82)	0.582 (0.713)
Punitive parenting	1.88 (1.24)	0.426 (0.806)
Warmth	0.52 (1.17)	0.380 (1.013)

CP = Conduct Problem; SE = Standard Error; PP = Psychopathic Traits

*p <.05

### Developmental outcomes (T4-T6)

Developmental outcomes of the different profiles have been compared across time considering behavioral and psychosocial adjustment variables (see Table 6 for total sample and Table 7 for High CP subsample). Parent-reported variables showed a clear tendency to be higher than those reported by teachers, except for bullying, which was reported in greater intensity by teachers, especially in total sample. All parent-reported variables remained significant (*p* <.05) for the total sample, with the Low prosociality + *PP* group standing out as having the highest levels of CP, social problems, emotional symptoms, hyperactivity, bullying, and victimization, along with the lowest levels of prosocial behavior, although differences between this group and the others emerged as significant just for CP, bullying, and victimization (with highest levels). The opposite trend is observed in the Normative development profile. For teacher-reported variables, the same trend was observed.

**Table 6:** ANOVA repeated measures for parent- (T4-T6) and teacher- (T4-T5) reported developmental outcomes in total sample

Total sample												
	Parent reported (T4-T6)						Teacher reported (T4-T5)					
	Normative development	Daring / Impulsive	Low prosoc + fear	Low prosoc + PP	F^b^(df = 3)	η²	Normative development	Daring / Impulsive	Low prosoc + fear	Low prosoc + PP	F^b^(df = 3)	η²
	M^a^ (SD)	M (SD)	M (SD)	M (SD)			M (SD)	M (SD)	M (SD)	M (SD)		
CP	1.40 (0.02)_a_	1.69 (0.04)_b_	1.72 (0.04)_b_	2.07 (0.06)_c_	62.71***	0.20	1.21 (0.02)_a_	1.51 (0.03)_b_	1.32 (0.03)_c_	1.70 (0.06)_d_	38.43***	0.11
SDQS	0.21 (0.01)_a_	0.22 (0.02)_a_	0.31 (0.02)_b_	0.35 (0.04)_b_	9.04*	0.03	0.16 (0.01)_a_	0.25 (0.02)_b_	0.21 (0.02)_b_	0.35 (0.03)_c_	11.73***	0.04
SDQP	1.83 (0.01)_b_	1.80 (0.03)_b_	1.60 (0.02)_a_	1.54 (0.03)_a_	58.86***	0.19	1.65 (0.01)_c_	1.52 (0.03)_b_	1.60 (0.03)_c_	1.42 (0.04)_a_	13.76***	0.04
SDQE	0.45 (0.02)_a_	0.46 (0.04)_a, b,c_	0.55(0.03)_b, c_	0.57 (0.05)_c_	3.53*	0.01	0.28 (0.04)_a_	0.29 (0.02)_a_	0.31 (0.02)_a_	0.36 (0.04)_a_	1.87	0.01
SDQH	0.50 (0.02)_a_	0.93 (0.04)_b_	0.76 (0.04)_c_	1.06 (0.06)_b_	56.78***	0.18	0.39 (0.02)_a_	0.76 (0.04)_c_	0.51 (0.04)_b_	0.81 (0.06)_c_	36.18***	0.11
Bullying	1.16 (0.02)_a_	1.30 (0.03)_b_	1.39 (0.03)_c_	1.62 (0.04)_d_	50.50***	0.17	1.25 (0.02)_a_	1.57 (0.04)_c_	1.39 (0.04)_b_	1.77 (0.06)_d_	33.50***	0.10
Victim	1.44 (0.05)_a_	1.59 (0.05)_b_	1.53 (0.05)_a_	1.74 (0.08)_c_	6.52**	0.03	1.14 (0.02)_a_	1.34 (0.03)_b_	1.19 (0.03)_a_	1.44 (0.05)_d_	21.50**	0.07

PP = Psychopathic Traits; M = Mean Z score; SD = Standard Deviation; SDQS = Strengths & Difficulties Questionnaire Social; SDQP = Strengths & Difficulties Questionnaire Prosocial; SDQE = Strengths & Difficulties Questionnaire Emotional; SDQH = Strengths & Difficulties Questionnaire Hyperactivity. Different subscripts (a, b, c, d) refer to significant differences between classes (*p* <.05) with post hoc Bonferroni test for multiple pairwise comparisons

^a^Mean values represent overall estimates of each analyzed variable across profiles

^b^*F* values represent between-subjects effect tests

*p <.05. ***p <.001

**Table 7:** ANOVA repeated measures parent (T4-T6) and teacher (T4-T5) reported in high CP subsample

High CP sample										
	Parent reported					Teacher reported				
	Daring / Impulsive	Low prosociality + fear	Low prosociality PP	F^b^ (df = 2)	η²	Daring / Impulsive	Low prosociality + fear	Low prosociality PP	F^b^ (df = 2)	η²
	M^a^ (SD)	M (SD)	M (SD)			M (SD)	M (SD)	M (SD)		
CP	2.58 (0.15)_a_	2.19 (0.12)_a_	2.53 (0.18)_a_	2.69	0.10	1.80 (0.12)_a_	1.68 (0.12)_a_	2.07 (0.19)_a_	1.61	0.04
SDQS	0.35 (0.07)_a_	0.38 (0.06)_a_	0.57 (0.09)_a_	2.09	0.08	0.29 (0.05)_a_	0.27 (0.04)_a_	0.63 (0.07)_b_	10.59***	0.23
SDQP	1.67 (0.07)_a_	1.51 (0.06)_a_	1.42 (0.09)_a_	2.72	0.10	1.45 (0.06) _b_	1.46 (0.06)_b_	1.17 (0.10)_a_	3.49*	0.10
SDQE	0.73 (0.09)_a_	0.61 (0.07)_a_	0.51 (0.11)_a_	1.14	0.04	0.34 (0.06)_a_	0.32 (0.05)_a_	0.51 (0.09)_a_	1.78	0.05
SDQH	1.37 (0.10)_b_	0.86 (0.09)_a_	0.13 (0.14)_a, b_	7.32*	0.25	0.92 (0.10)_a_	0.78 (0.10)_a_	1.30 (0.16)_b_	3.76*	0.10
Bullying	1.68 (0.12)_a_	1.67 (0.10)_a_	2.00 (0.15)_a_	1.91	0.07	1.88 (0.12)_a_	1.61 (0.12)_a_	1.96 (0.19)_a_	1.80	0.05
Victimiz	1.76 (0.17)_a_	1.82 (0.14)_a_	2.09 (0.21)_a_	0.83	0.03	1.41 (0.07)_a_	1.26 (0.07)_a_	1.75 (0.11)_b_	7.61*	0.18

CP = Conduct Problem; PP = Psychopathic Traits; M = Mean Z score; SD = Standard Deviation; SDQS = Strengths & Difficulties Questionnaire Social; SDQP = Strengths & Difficulties Questionnaire Prosocial; SDQE = Strengths & Difficulties Questionnaire Emotional; SDQH = Strengths & Difficulties Questionnaire Hyperactivity. Different subscripts (a, b) refer to significant differences between classes (*p* <.05) with post hoc Bonferroni test for multiple pairwise comparisons

^a^Mean values represent overall estimates of each analyzed variable across profiles

^b^*F* values represent between-subjects effect tests

*p <.05. ***p <.001

Values reported for the High CP group were also, as a rule, higher than those reported for the total sample group, regardless of whether they were reported by parents or by teachers. Considering parent-reported measures, only hyperactivity levels remained significant, distinguishing only between the Daring/Impulsive and Low prosociality + fear groups, with the latter showing the highest values. In teacher-reported variables, however, notable differences arose, with the Low prosociality + *PP* profile showing the highest values in social problems, hyperactivity symptoms, and victimization, and the lowest in prosocial behavior.

## Discussion

This study explored the heterogeneity of CP throughout childhood, based on relevant variables (individual and family-related) assessed through a longitudinal study. Previous research in this area has typically employed a variable-centered approach [33, 34], confirming the significance of several key domains in conceptualizing heterogeneity in CP: ODD (characterized by high levels of negative emotionality, anger/frustration), ADHD (low effortful control, attention deficits, impulsivity), and CU traits (low guilt and empathy, increased CP and aggression) [28]; each of one associated with specific behavioral manifestations [93]. In this context, the aim of the present study was to evaluate whether a person-centered analytical approach aligns with these findings, including the influence of some predictors and outcomes for each profile.

### Identifying resulting profiles

This study analyzed two distinct samples: a broad community-based sample and a subsample characterized by high levels of CP (1.5 *SD* above the mean). The results identified four distinct profiles in the total sample and three profiles in the High CP group, in line with the hypotheses of heterogeneity in CP. This discrepancy between samples was expected, as the missing group in the High CP subsample corresponds to the normative development group, which includes most of the children from the total sample (58.2%). Among the remaining three groups, the two profiles characterized by low prosociality displayed similar profiling trends across both samples, while the other, Daring/Impulsive, differed in the clustering and intensity of some (psychopathic) traits.

Of the identified groups, two, Daring/Impulsive (characterized by high daring and fearlessness) and Low prosociality + *PP* (notable for pronounced CU traits and low prosociality) are consistent with the ADHD and CU domains described by Waller et al. [28] in both samples. Furthermore, results for the Low prosociality + *PP* profile revealed that children in this group exhibited not only high CU traits but also elevated fearlessness and the two additional psychopathic traits: INS and GD [94]. This finding is significant, as it highlights the importance of considering all psychopathic traits—not solely CU traits—for a more accurate understanding of this profile [95, 96]. This group (with all three psychopathic traits) is associated with an increased risk of externalizing problems and stable, long-term antisocial behavior [65].

The third emergent group, Low prosociality + fear, does not align with the established characteristics of the ODD profile. Negative emotionality, a central feature of the ODD domain, was consistently elevated across all three profiles in the High CP subsample. This suggests that negative emotionality may serve as a common trait across CP, supporting some transdiagnostic theoretical frameworks [97, 98]. In this study, the Low Prosociality + fear profile is characterized by low prosocial behavior, heightened fear, low daring, and significant differences in anxiety compared to the other groups. Additionally, this group exhibited elevated psychopathic traits, suggesting a potential link to the *acquired CU variant*. This variant posits that CU traits may develop through the interaction of environmental factors, with anxiety playing a central role in this process [99].

Regarding gender differences, several key findings emerge. First, while boys and girls are similarly represented in the problematic profiles of the total sample, boys are more frequently found in the profiles within the High CP subsample, particularly in those characterized by low prosociality. This observation is consistent with the trend that boys tend to exhibit higher levels of CP [22], which results in 70.2% of this subsample being boys, leading to an underrepresentation of girls. Future studies should consider gender differences and, when appropriate, include girls who score above their reference group (rather than in the pooled sample) to ensure proper representation. This approach is relevant because, although CP scores are generally higher in boys, a girl with relatively lower CP scores may still exhibit behaviors that deviate significantly from her reference group, which could be problematic [22, 100]. The relatively small gender differences observed in the Daring/Impulsive profile within the High CP subsample (62.7% boys vs. 37.3% girls) could have significant implications that merit further investigation. CP in girls may not primarily manifest as low prosociality but rather through different patterns, such as negative emotionality, daring, impulsivity, and fearlessness. This suggests that, particularly for girls, intervention and treatment strategies should focus on these aspects rather than exclusively on prosociality.

### Relevant individual and parenting variables for profile membership

Contrary to expectations, positive parenting and its core element, warmth, do not significantly influence group membership in any profile or sample [50, 51]. However, punitive parenting emerges as a key distinguishing factor in the total sample. While it does not differentiate between the two low prosociality profiles, higher levels predict membership in either of these profiles compared to the normative group. For the Low prosociality + *PP* profile, higher punitive parenting also differentiates this group from the Daring/Impulsive Profile in the total sample. Similarly, inconsistent parenting predicts membership in the Low Prosociality + *PP* profile rather than the normative group. This aligns with existing literature underscoring the critical role of punitive and inconsistent parenting in shaping outcomes for this group [52, 54], as these styles are consistently linked to higher levels of CP [31, 101]. Beyond parenting variables, a shared predictor for both low prosociality profiles is a deficit in prosocial and communicative skills, which distinguishes these groups from the normative and daring/impulsive profiles. This finding aligns with research connecting psychopathic traits to socio-communicative impairments [102, 103].

In the High CP subsample, a few variables differentiate between profiles, reflecting the shared risk factors across the externalizing spectrum. Nevertheless, two variables stand out: inconsistent parenting and activity levels. Increased inconsistent parenting predicts membership in the Daring/Impulsive profile compared to the Low prosociality + fear profile, reinforcing the importance of parenting in this group. This finding diverges from some recent studies suggesting less relevance of parenting for Low prosociality + fear group [104]. Activity levels consistently differentiate the Low prosociality + fear profile, characterized by lower levels, from the Daring/Impulsive profile across both samples. This suggests a stronger association with the hyperactivity component of ADHD in the Daring/Impulsive profile [105].

The reciprocal influence between temperamental variables and parenting practices is particularly important, as we know that parenting strategies can moderate the relationship between temperament and CP [106]. Additionally, we also know that certain temperamental characteristics promote parenting styles characterized by harshness [107]. Moreover, specific temperamental traits for each domain, when combined with negative parenting strategies, not only increase the severity of symptoms but also lead to a higher number of CP. For example, low effortful control is amplified in the context of harsh parenting [108]; high surgency combined with negative parenting results in greater symptom severity of ODD [109]; and fearlessness in a harsh parenting context increases CU traits and future CP [110, 111], whereas the opposite trend is found for ODD, with high fear and harsh parenting leading to more ODD symptoms [111].

### Developmental outcomes

In general, parents report higher intensity of outcomes, whereas information of teachers identify more significant differences between profiles. More CP [64] and bullying [59] are observed in the Low prosociality + *PP* profile in the total sample, closely associated with the domains of the “cold pathway” proposed by Waller et al. [28]. Additionally, this group consistently exhibits the highest scores, according to both parent and teacher reports, in peer problems, low prosociality, emotional issues, and hyperactivity. These results confirm it as the profile with the highest risk and poorest prognosis [31].

In the High CP group, fewer behavioral attributes remain significant, which may reflect the shared behavioral characteristics commonly observed within CP groups [44]. Among the significant variables, hyperactivity does not appear to be a distinguishing trait for the Daring/Impulsive compared to the Low prosociality + *PP* profile, with the latter showing higher levels of hyperactivity. This finding supports the idea that when the Low prosociality + *PP* profile includes hyperactivity traits, it may represent a more stable and severe behavioral trajectory [112]. Moreover, the Low prosociality + *PP* profile consistently demonstrates a higher number of peer problems and lower levels of prosocial behavior, reinforcing its distinctive features [113, 114]. Results about victimization highlight the Low prosociality + *PP* profile as the group with the highest victimization levels, a consistent finding across both samples and reporters. While traditionally associated with bullying behaviors [59], recent research also emphasizes this group’s vulnerability to victimization [68].

### Implications

Current findings underscore the heterogeneity inherent to CP, emphasizing the critical importance of considering the distinctive characteristics of each identified profile, as well as their commonalities, which is essential in both theory and practice. Theoretically, it is important to consider this heterogeneity and to pursue further research that accounts for the specific characteristics of each CP profile. To this end, a more systematic framework to promote the integration of phenotypic, etiological and developmental levels of explanation is particularly needed [115]. Practically, it is essential to tailor and implement target psychosocial interventions based on distinct CP profiles. Early intervention is critical, as it leads to the best outcomes [116] and fostering well-being and school adjustment [117]. Even combinations that are more resistant to conventional treatments (high CP and CU traits) should be considered for its worst future prognosis [97]. Without specialized treatment, negative behaviors such as aggression, harm, bullying, and CU traits may become stable over time [54]. Intervention and particularly prevention programs also benefit from targeting shared risk factors underlying different subtypes of CP. For instance, parenting programs remain to be effective in targeting child CP [118], also for more resistant subgroups (i.e., high on CU traits [119]), with proven effectiveness after tailoring their specific needs [120]. It overall underscores the importance of clearly identifying distinctive phenotypical configurations of child CP, accounting for both common and specific underlying mechanisms, which may positively impact the effect of evidence-based applied interventions.

### Strengths, limitations and future directions

These results have several limitations. First, the community sample limits the identification of pure clinical constructs, leading to a small CP group with low sample sizes across profiles. Second, some subscales, such as Sociability and Punitive Practices, had moderate-low reliability. Yet, MIC values, less dependent on the number of items, provided additional support for their internal consistency. Also, the EAS is a widely used instrument for assessing sociability, and the APQ-Pr is a well-established measure of punitive practices, both of which have been validated in the Spanish context [78, 121]. Nevertheless, these results should be interpreted with caution as low reliability could undermine the established conclusions. Third, although a multi-informant approach was included for assessing developmental outcomes, the effect of shared method variance should not be discarded as only parent-reported variables were used for latent profile indicators and predictors. Fourth, key variables for profile identification (e.g., irritability, aggression, emotion recognition) were not included, which may have restrained the identification of a more ODD-based group. Finally, sample attrition should be noted as it may affect, to some extent, the results. Even though the causes of attrition were similar than those commonly observed in longitudinal studies [71], when longitudinal research span different developmental periods, the impact of other variables, including SES and different developmental factors (e.g., IQ, executive function, pregnancy and birth problems) should be also considered [122]. Additional efforts to promote participants’ retention and to mitigate the potential impact of attrition should be particularly encouraged. The study’s strengths include its longitudinal design, use of a stringent CP cutoff to select prominent cases, multiple informants (parents and teachers), and the use of a person-centered approach to profile CP groups. Future research should build on these strengths, incorporating children into their own evaluation process. This will provide a clearer understanding of the developmental heterogeneity of child CP. Also, future studies should explore the stability of temperamental and psychopathic trait profiles over time. Future research will also benefit from the inclusion of additional outcomes, relevant in adolescent adjustment (e.g., academic performance, antisocial behavior, criminal activity), that will help to further understand how the different profiles develop across different developmental stages as well as their impact in the long-term. Finally, it would be important to examine the impact of gender on these latent profiles. Although we did not explore this in the current study, related research using the ELISA sample has identified gender-related differences in outcomes associated with the same latent profiles, which should be further investigated (for more details, see [123]).

## Conclusions

This manuscript highlights the usefulness of a person-centered approach in analyzing the heterogeneity of CP. Based on a well-established heterogeneity model, which postulates the existence of three distinct domains with specific characteristics—ODD, ADHD, and CU—these domains were tested using a community sample and a High CP subsample. The different profiles identified in both samples align with the ADHD and CU groups previously proposed. Among the key predictors of group membership, different parenting styles stand out (e.g., inconsistent parenting). In contrast, regarding behavioral trajectories, the *Low Prosociality + PP Traits* profile (aligned with the CU domain) emerges as the most severe in terms of behavioral risk. The identification of these distinct profiles holds significant theoretical and practical implications, fostering further research and improving clinical interventions tailored to the specific characteristics of each profile.

## Electronic supplementary material

Below is the link to the electronic supplementary material.

**Supplementary Material 1: Appendix**. Information related to variables used in the study. This section contains information on the variables used in the study, their main descriptive statistics, their use according to the different waves of time and the informants who reported them.

## Data Availability

Data and materials that support the findings of this study are available from the corresponding author, B.D.-V., upon request.

## Abbreviations

CP: Conduct problems
ODD: Oppositional defiant disorder
ADHD: Attention-deficit/hyperactivity disorder
CU: Callous-unemotional
NW: Northwest
PP: Psychopathic traits
CBCL: Child Behavioral Checklist
GD: Grandiose-deceitful
INS: Impulsive-need of stimulation
CADS-P: Child and Adolescent Dispositions Scale
CPTI: Child Problematic Traits Inventory
EAS TS-C: EAS Temperament Survey for Children
APQ-Pr: Alabama Parenting Questionnaire- Preschool revision
SDQ: Strengths and Difficulties Questionnaire
LPA: Latent profile analysis
BIC: Bayesian information criterion
LMR: Lo-Mendell-Rubin

## Footnotes

1 For those variables with poor Cronbach’s alpha (> 0.60), the mean inter-item correlation (MIC) was computed as an additional indicator of internal consistency, with scores ranging 0.15-0.50 being considered adequate [74]. Scales affected were T1 Sociability (MIC = 0.31) and Punitive parenting (MIC = 0.32).

2 See http://www.personalitydevelopmentcollaborative.org/project-page-elisa/ for more details.
