# Supplementary material for: Heterogeneity of early-onset conduct problems: assessing different profiles, predictors and outcomes across childhood
Source: Child Adolesc Psychiatry Ment Health. 2025 Apr 16;19:43. doi: 10.1186/s13034-025-00897-2 (PMC12004637; doi:10.1186/s13034-025-00897-2)
Supplement: Supplementary file 1 — Supplementary Material 1: Appendix. Information related to variables used in the study. This section contains information on the variables used in the study, their main descriptive statistics, their use according to the different waves of time and the informants who reported them. [file 13034_2025_897_MOESM1_ESM.docx]

**Appendix**

**Information related to variables used in the study**

Table S1_ *Variables Used in the Analysis*

Table S2_ *Descriptive Statistics of the Main Study Variables*

**Table S1**

*Variables Used in the Analysis*

| **Aim** | | **Type and Informant** | **Variable** | |
| --- | --- | --- | --- | --- |
| **Children variables used for Latent Profile indicators (T3)** | Temperament variables– PR | | | Negative emotionality  Prosociality  Daring  Fearlessness |
|  | Personality variables (psychopathic traits)– PR | | | Grandiose-Deceitful  Impulsive-Need for stimulation  Callous-Unemotional traits |
| **Variables for further definition (T3)** | Behavioural variables - PR | | | ODD, ADHD, ANX |
| **Predictor variables (T1)** | Children´s temperament variables– PR | | | Emotionality, sociability, shyness & activity  Social competence |
|  | Parenting variables– PR | | | Parental warmth  Positive parenting  Inconsistent parenting  Punitive parenting |
| **Longitudinal outcomes (T4-T6)** | Children´s variables – PR (T4-T6) & TR (T4-T5) | | | Conduct problems  Hyperactivity  Prosocial behaviour  Emotional symptoms  Peer problems  Bullying  Victimization |

*Note.* T = Time; PR = Parent- Reported; TR = Teacher Reported; ODD = Oppositional Defiant Disorder; ADHD = Attention-deficit/hyperactivity disorder; ANX = Anxiety.

**Table S2**

*Descriptive Statistics of the Main Study Variables*

| **Variable** | | **M(SD)** | | | | | | | | |
| --- | --- | --- | --- | --- | --- | --- | --- | --- | --- | --- |
|  |  | **Total Sample** | | | |  | **High CP group (1.5SD)** | | | |
| **Latent Profile indicators (T3)** | Negative emotionality  Prosociality  Daring  Fearlessness | 2.12 (0.60)  3.27 (0.56)  1.88 (0.72)  1.56 (0.60) | | | |  | 3.01 (0.59)  2.90 (0.63)  2.44 (0.80)  2.10 (0.71) | | | |
|  | GD  INS  CU | 1.36 (0.45)  2.07 (0.60)  1.32 (0.43) | | | |  | 1.92 (0.60)  2.73 (0.51)  1.86 (0.57) | | | |
| **Variables for further definition (T3)** | ODD  ADHD  ANX | 0.47 (0.40)  0.52 (0.42)  0.38 (0.30) | | | |  | 1.08 (0.38)  1.02 (0.44)  0.59 (0.34) | | | |
| **Predictors (T1)** | Emotionality  Sociability  Shyness  Activity  Emotion regulation  Prosocial & Commun. skills | 3.03 (0.81)  3.53 (0.63)  2.90 (1.07)  3.69 (1.00)  1.70 (0.63)  2.38 (0.70) | | | |  | 3.64 (0.77)  3.45 (0.72)  2.74 (1.19)  4.18 (4.18)  1.18 (0.56)  1.90 (0.64) | | | |
|  | Parental warmth  Positive parenting  Inconsistent parenting  Punitive parenting | 4.70 (0.39)  4.44 (0.35)  1.98 (0.50)  1.71 (0.41) | | | |  | 4.59 (0.45)  4.32 (0.40)  2.20 (0.50)  1.93 (0.45) | | | |
| **Longitudinal outcomes** |  | **Parent-reported (T4-T5-T6)** | | | | | | | | |
|  |  | **T4** | **T5** | | **T6** |  | **T4** | **T5** | | **T6** |
|  | Conduct problems  Hyperactivity  Prosocial behavior  Emotional symptoms  Peer problems  Bullying  Victimization | 1.55 (0.49)  0.70 (0.50)  1.75 (0.29)  0.45 (0.42)  0.23 (0.30)  1.31 (0.40)  1.55 (0.66) | 1.44 (0.45)  0.68 (0.51)  1.75 (0.29)  0.48 (0.42)  0.24 (0.31)  1.25 (0.36)  1.53 (0.68) | | 1.35 (0.40)  0.62 (0.50)  1.77 (0.28)  0.48 (0.42)  0.26 (0.32)  1.23 (0.37)  1.49 (0.67) |  | 2.37 (0.63)  1.19 (0.49)  1.51 (0.31)  0.63 (0.46)  0.41 (0.36)  1.80 (0.57)  1.87 (0.78) | 2.20 (0.65)  1.14 (0.53)  1.47 (0.38)  0.69 (0.41)  0.45 (0.38)  1.73 (0.56)  1.97 (0.76) | | 2.00 (0.63)  1.12 (0.53)  1.59 (0.33)  0.64 (0.46)  0.44 (0.39)  1.69 (0.65)  1.80 (0.77) |
|  |  | **Teacher-reported (T4-T5)** | | | | | | | | |
|  |  | **T4** | | **T5** | |  | **T4** | | **T5** | |
|  | Conduct problems  Hyperactivity  Prosocial behavior  Emotional symptoms  Peer problems  Bullying  Victimization | 1.32 (0.51)  0.53 (0.54)  1.56 (0.41)  0.30 (0.37)  0.31 (0.30)  1.35 (0.56)  1.20 (0.40) | | 1.31 (0.49)  0.48 (0.52)  1.65 (0.40)  0.29 (0.35)  0.19 (0.30)  1.36 (0.57)  1.21 (0.44) | |  | 1.88 (0.77)  0.99 (0.65)  1.35 (0.43)  0.37 (0.40)  0.33 (0.33)  1.84 (0.74)  1.42 (0.51) | | 1.69 (0.72)  0.84 (0.60)  1.47 (0.48)  0.39 (0.39)  0.33 (0.38)  1.67 (0.80)  1.38 (0.52) | |

*Note.* M = mean, SD = Standard Deviation, GD = Grandiose-Deceitful, INS = Impulsive-Need for stimulation, CU = Callous-Unemotional traits

To calculate the High CP group, parent-reported CP scores at T3 were used: *M* = 2.74, *SD* = 0.38.
